# Predictive Potential of ECMO Blood Flow for Hemolysis and Outcome of Patients with Severe ARDS

**DOI:** 10.3390/jcm14010140

**Published:** 2024-12-29

**Authors:** Victoria Bünger, Martin Russ, Wolfgang M Kuebler, Mario Menk, Steffen Weber-Carstens, Jan A Graw

**Affiliations:** 1 Department of Anesthesiology and Intensive Care Medicine CCM/CVK Charité—Universitätsmedizin Berlin, Corporate Member of Freie Universität Berlin, Humboldt-Universität zu Berlin, 13353 Berlin, Germany; 2 ARDS/ECMO Centrum Charité, Charité—Universitätsmedizin Berlin, 13353 Berlin, Germany; 3 Institute of Physiology, Charité—Universitätsmedizin Berlin, 10117 Berlin, Germany; 4 Department of Anesthesiology and Intensive Care Medicine, University Hospital “Carl Gustav Carus”, Technische Universität Dresden, 01069 Dresden, Germany; 5 Department of Anesthesiology and Intensive Care Medicine, Universitätsklinikum Ulm, Ulm University, 89081 Ulm, Germany

**Keywords:** blood flow, hemolysis, extracorporeal membrane oxygenation, acute lung injury

## Abstract

**Background:** Treatment with veno-venous extracorporeal membrane oxygenation (VV ECMO) has become a frequently considered rescue therapy in patients with severe acute respiratory distress syndrome (ARDS). Hemolysis is a common complication in patients treated with ECMO. Currently, it is unclear whether increased ECMO blood flow (Q̇_EC_) contributes to mortality and might be associated with increased hemolysis. **Methods:** A total of 441 patients with ARDS and VV ECMO, treated in a tertiary ARDS center, were included. The Q̇_EC_ value for a significant increase in ICU mortality was determined by binary recursive partitioning. Linear regression analysis was performed to analyze a correlation between mean Q̇_EC_ and mean plasma concentrations of cell-free hemoglobin (CFH). **Results:** A Q̇_EC_ of 4 L/min divided the cohort into two groups with significantly different ICU mortality (Q̇_EC_ ≤ 4 L/min: 39.3% (*n* = 300) versus Q̇_EC_ > 4 L/min: 71.6% (*n* = 141), *p* < 0.001). Patients with Q̇_EC_ > 4 L/min had a higher 28-day mortality. Furthermore, a higher mean Q̇_EC_ was associated with increased CFH and decreased haptoglobin plasma concentrations. **Conclusion:** In patients with ARDS and VV ECMO, a mean Q̇_EC_ > 4 L/min is associated with increased mortality, increased CFH and decreased haptoglobin plasma concentrations. Whether increased hemolysis determines the poorer outcome associated with higher Q̇_EC_ should be the subject of future research.

## 1. Background

In recent years, venous–venous extracorporeal membrane oxygenation (VV ECMO) has evolved as a rescue therapy for patients with a severe acute respiratory distress syndrome (ARDS) with refractory hypoxemia, despite lung protective ventilation and conservative therapy, including prone positioning [1,2]. Thus, the latest guidelines of the American Thoracic Society (ATS) and the European Society of Intensive Care Medicine (ESICM) suggest to consider the application of VV ECMO in patients who suffer from refractory hypoxemia, based on the inclusion criteria of the EOLIA trial [3,4]. At the same time, VV ECMO provides an opportunity to facilitate lung protective ventilation, even in patients with a severity of disease which negates mechanical ventilation within lung protective ventilation pressures and frequencies [5]. In order to support gas exchange with VV ECMO, the drainage of venous blood through one or two large bore cannulas, a consecutive pump-driven passage through a membrane oxygenator and the return through another large bore venous cannula is necessary [6].

Hemolysis is a common complication in patients treated with extracorporeal organ replacement systems, particularly in systems using membrane technology for gas exchange, like VV and veno-arterial (VA) ECMO [7,8,9]. Hemolysis has been reported in up to 41% of patients treated with VV ECMO [10,11,12]. Notably, patients receiving VA ECMO therapy for extracorporeal cardiac support show higher incidences and degrees of hemolysis compared to patients treated with VV ECMO [7,13,14,15]. Increased plasma concentrations of cell-free hemoglobin (CFH) are known to be associated with increased mortality and organ dysfunction, such as renal failure in patients with severe ARDS, and therapy with VV ECMO [16,17]. In contrast, increased plasma concentrations of the CFH-scavenger haptoglobin seem to have a protective effect [17,18,19,20,21].

To date, there is conflicting evidence on the association of increased ECMO blood flow (Q̇_EC_) and increased hemolysis. While a significant increase in CFH plasma concentration was noted in patients treated with an Q̇_EC_ ≥ 3 L/min by Lehle and colleagues, other data reported no association between Q̇_EC_ and hemolysis [22,23]. The combined analysis of retrospective patient data with computational fluid dynamics suggests that targeting low pump pressures and the lowest possible circuit resistance reduces trauma to the red blood cells, irrespective of the Q̇_EC_ [24]. However, multifactorial causes influence pump pressures and overall circuit resistance in the clinical situation, mainly Q̇_EC_, the available circuits and feasible vascular access.

In this study, we hypothesized that overall higher Q̇_EC_s are associated with an increased mortality in patients with ARDS treated with VV ECMO compared to patients with ARDS and treated with VV ECMO and lower Q̇_EC_s. Therefore, the primary objective of this study was to identify a threshold for a mean Q̇_EC_ that is associated with increased mortality. Furthermore, the data were analyzed to evaluate whether an increased Q̇_EC_ and increased plasma concentrations of CFH were independently associated with mortality in ARDS patients treated with VV ECMO.

## 2. Methods

### 2.1. Study Design and Setting

The study collective comprised a previously analyzed cohort of patients with ARDS admitted to a tertiary ARDS/ECMO referral center in Berlin, Germany, between January 2007 and December 2018 [16]. All patients with ARDS and treatment with VV ECMO, documented blood flow data and measured plasma concentrations of CFH and haptoglobin were included in the analysis. The retrospective analysis was approved by the Medical Ethics Committee of the Charité–Universitätsmedizin Berlin (No. EA2/019/19).

### 2.2. Participants and Data Sources

Eligible patients were grouped according to a calculated cut-off value for mean Q̇_EC_, as specified in the statistical analysis section. The exclusion criteria were as follows: patients with ARDS receiving no treatment with ECMO, treatment with veno-arterial or veno-veno-arterial ECMO and patients who died shortly after admission [18]. Patient data and laboratory values were extracted from two electronic patient data management systems used at the hospital (COPRA, Sasbachwalden, Germany and SAP, Walldorf, Germany). CFH and haptoglobin plasma concentrations were measured as described previously [17].

### 2.3. Statistical Analysis

The cut-off value for the mean Q̇_EC_ over the entire ECMO treatment time, regarding a significant increase in ICU mortality, was calculated by binary recursive partitioning. Baseline characteristics between the groups were compared. Differences in continuous variables were tested for significance using a Mann–Whitney-U test, and differences in frequencies were tested by a Fisher’s test as appropriate. A two-tailed *p* value < 0.05 was considered significant. All significantly different variables were included in a multivariate logistic regression model for ICU mortality. Simple logistic regression was performed for the mean Q̇_EC_ and the ICU mortality. Multivariable logistic regression was performed for mortality including the mean Q̇_EC_, and possible confounding variables and variance inflation factors (VIF) were calculated to assess multicollinearity. For the direct correlation of mean CFH and mean ECMO blood flow, a linear regression model was used. Statistical analysis was performed with R Version 4.4.0, generalized linear models (logistic) (“GLM”) were used for all logistic regressions and a linear model (“lm”) was used for the linear regression, both from the “stats” package.

## 3. Results

In total, 441 patients with ARDS and treatment with VV ECMO were included in the analysis. A simple logistic regression analysis for mortality revealed a significant association with mean Q̇_EC_ over ECMO treatment time (OR 1.99 [95% CI 1.60–2.49], *p* < 0.001). Considering Q̇_EC_ as a continuous variable, these data suggest that an increase in Q̇_EC_ by each additional liter per minute doubles a patient’s risk of death during intensive care unit (ICU) treatment.

**Figure 1:**
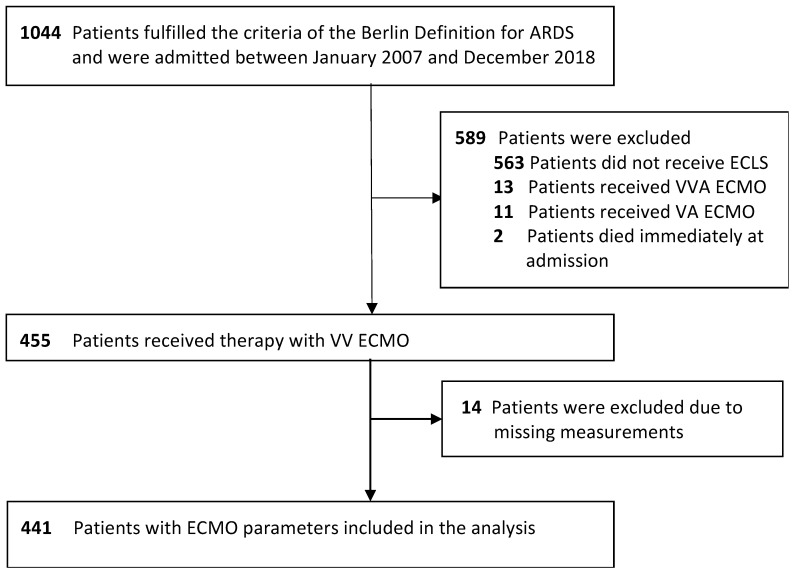
Patient flow chart.

Using recursive binary partitioning, a cut-off value for a mean Q̇_EC_ of 3.99 L/min, rounded to 4 L/min, was associated with a significant increase in ICU mortality (ICU mortality of 39.3% (*n* = 300) vs. 71.6% (*n* = 141) for Q̇_EC_ ≤ 4 L/min vs. Q̇_EC_ > 4 L/min, *p* < 0.001, Figure 1). Patients were grouped accordingly, and baseline characteristics were compared (Table 1). Groups differed significantly in age, gender, mean ECMO blood and gas flow over the treatment time, mean CFH and haptoglobin plasma concentrations, the sum of transfused units of packed red blood cells and ICU length of stay (Table 1). Patients did not differ in classification for ARDS severity (severe ARDS: 93.7% (*n* = 300) vs. 92.9% (*n* = 141) for Q̇_EC_ ≤ 4 L/min vs. Q̇_EC_ > 4 L/min, *p* = 0.704).

After including the relevant significantly different baseline characteristics in a multivariate regression model for ICU mortality, the mean Q̇_EC_, age, gender, mean ECMO blood and sweep gas flow and mean CFH and haptoglobin plasma concentrations remained significantly associated with mortality (Table 2). Testing for multicollinearity was conducted, and variance inflation factors (VIF) ranged between 1 and 1.5, indicating no significant collinearity.

In a linear regression analysis, a significant positive correlation between the mean Q̇_EC_ and the mean CFH plasma concentrations was observed (slope 3.009 [95% CI 0.92–5.1], *p* = 0.005).

## 4. Discussion

In a cohort of 441 patients with ARDS treated with VV ECMO, patients with a mean Q̇_EC_ greater than 4 l/min over the course of ECMO therapy had a significantly higher risk of death compared to patients with a mean Q̇_EC_ below 4 L/min. Furthermore, besides patients’ age, gender and mean Q̇_EC_, increased plasma concentrations of CFH, decreased plasma concentrations of haptoglobin and increased mean ECMO gas flow were independently associated with a higher mortality while a direct and significant correlation between mean Q̇_EC_ and mean CFH plasma concentration was observed.

In patients receiving VV ECMO for treatment of refractory hypoxemia in severe ARDS, oxygenation is determined by the respective blood flow over the ECMO membrane in proportion to a patient’s cardiac output and the remaining ability of the lung for native gas exchange. Therefore, patients requiring a higher Q̇_EC_ to maintain appropriate systemic oxygenation tend to either suffer from severe deprivations of pulmonary gas exchange or a high oxygen demand as well as cardiac output due to sepsis. Despite this, these patients can be considered to be sicker compared to patients requiring lower Q̇_EC_. The ICU severity scores APACHE II, SAPS2, and SOFA at admission to the ICU did not differ between the groups of the investigated cohort (Table 1). Furthermore, the severity of ARDS on admission, characterized by gas exchange and parameters of mechanical ventilation, such as PEEP or driving pressure, did not differ between groups. Given these findings, and considering that analyses were performed with mean Q̇_EC_ over therapy time, the data suggest that patients with a higher mean Q̇_EC_ had a more complicated ICU course, or required longer therapy time for the resolution of the ARDS.

The current investigation does not support a differentiation between the effect of increasing Q̇_EC_s and the effect of the overall burden of disease over time on mortality. Whether the higher CFH plasma concentrations resulted from the exposure of red blood cells to increased mechanical stress with higher Q̇_EC_ or from the severity of the underlying disease remains unclear from these retrospective data. However, the findings in this relatively large cohort of 441 patients with ARDS treated with VV ECMO suggest a linear association between Q̇_EC_ and the plasma concentration of CFH which cannot be explained by the course of the underlying disease. Whether hemolysis itself is the main contributor to increased mortality observed with higher blood flow remains speculative. In fact, in our cohort, each increase of one liter per minute in ECMO blood flow was associated with an increase in mortality with the factor 1.99.

While increased plasma concentrations of CFH are associated with increased mortality and organ dysfunction in patients with ARDS and treatment with VV ECMO, previous data underscores that also plasma concentrations of haptoglobin need to be taken into consideration when CFH plasma concentrations are evaluated [12,16,17,18,25]. With appropriate haptoglobin plasma concentrations, higher CFH plasma concentrations can be tolerated given that haptoglobin is the natural CFH scavenger and facilitates macrophagic internalization of CFH with consecutive removal from plasma [26]. Consequently, like CFH plasma concentrations, haptoglobin plasma concentrations were independently associated with mortality and Q̇_EC_ in the investigated cohort. The experimental data suggest a beneficial effect of therapy with exogenous haptoglobin in conditions of increased hemolysis [27,28,29]. Whether patients with ARDS and treatment with VV ECMO requiring high Q̇_EC_s might serve as a relevant target group for a clinical application of this therapeutic approach is currently unknown.

Limited by the retrospective and single-center design, the various reasons for Q̇_EC_ adjustment remain unclear from the obtained and reported data. Besides adjustments of Q̇_EC_ for improvement of oxygenation or sings for inappropriate oxygen carrying capacity of the blood, bleeding events and comorbidities requiring appropriate systemic coagulation might interfere with the decisions for adjustments of Q̇_EC_. When systemic anticoagulation should be omitted in cases of e.g. intracranial hemorrhages which can occur in around 10% of patients treated with VV ECMO, usually higher Q̇_EC_ is targeted to protect the function of the oxygenator and prevent clotting within the gas transfer membrane [30,31,32]. A multiple regression analysis was applied to correct for confounders. However, this approach might still not have completely covered the entirety of all possible factors contributing to mortality in patients with ARDS and treatment with VV ECMO, which clearly presents one of the sickest ICU patient populations. After all, these data might be useful for planning future prospective clinical studies addressing the relevance of Q̇_EC_ and hemolysis in patients with treatment with VV ECMO.

## 5. Conclusions

In patients with ARDS and treatment with VV ECMO, a mean Q̇_EC_s greater than 4 L/min was associated with increased mortality, compared to a mean Q̇_EC_s below 4 L/min. In addition, high mean Q̇_EC_s were associated with increased hemolysis and decreased haptoglobin plasma concentrations suggesting relevant hemolysis with higher mean Q̇_EC_s. Whether increased hemolysis with higher Q̇_EC_s determines the poorer outcome and whether higher plasma concentrations of haptoglobin might have a protective effect should be the subject of future research.

## Figures and Tables

**Table 1:** Comparison of baseline characteristics between the groups.

Characteristic	Q̇_EC_ ≤ 4 L/min (*n* = 300)	Q̇_EC_ > 4 L/min (*n* = 141)	*p* Value
Age, years [IQR]	50.00 [38.00, 63.00]	47.00 [35.00, 56.00]	0.021
Male sex, n (%)	182 (60.7)	102 (72.3)	0.019
Body mass index, kg/m^2^ [IQR]	24.82 [21.91, 29.39]	26.12 [22.00, 30.84]	0.441
Charlson comorbidity index [IQR]	2.00 [1.00, 4.00]	2.00 [1.00, 4.00]	0.241
SOFA at ARDS onset [IQR]	12.00 [9.00, 15.00]	12.00 [9.00, 16.00]	0.272
APACHE at ARDS onset [IQR]	27.50 [22.00, 34.00]	27.00 [21.00, 35.00]	0.968
SAPS at ARDS onset [IQR]	56.50 [42.00, 70.00]	57.00 [37.00, 71.00]	0.825
RASS at ARDS onset [IQR]	−5.00 [−5.00, −4.00]	−5.00 [−5.00, −4.00]	0.840
Pulmonary origin, *n* (%)	271 (90.3)	123 (87.2)	0.325
ARDS etiology, *n* (%)			0.199
Pneumonia	215 (71.7)	90 (63.8)	
Aspiration	23 (7.7)	16 (11.3)	
Other	62 (20.7)	35 (24.8)	
External ventilation, days [IQR]	2.00 [1.00, 5.00]	2.00 [1.00, 6.00]	0.677
PaO_2_/FiO_2,_ mmHg [IQR]	66.7 [53.3, 85.7]	65.6 [54.1, 86.5]	0.847
PaO_2_, mmHg [IQR]	65.2 [52.7, 80.3]	64.8 [53.2, 83.1]	0.744
PaCO_2_, mmHg [IQR]	64.4 [52.2, 81.2]	66.7 [56.6, 84.9]	0.147
PIP, cmH_2_O [IQR]	39 [35, 43]	41 [35, 45]	0.173
Pplat, cmH_2_O [IQR]	36 [32, 41]	36.00 [33, 39]	0.967
PEEP, cmH_2_O [IQR]	19 [15, 21]	18 [14, 21]	0.387
Driving pressure, cmH_2_O [IQR]	18 [14, 21]	17 [15, 21]	0.745
Tidal volume/PBW, mL/kg [IQR]	5.65 [4.36, 6.95]	5.85 [4.41, 7.28]	0.690
Respiratory rate, breaths/min [IQR]	24.77 [20.72, 27.38]	24.00 [20.75, 27.82]	0.671
Compliance, mL/cmH_2_O [IQR]	29.00 [19.86, 43.39]	32.00 [22.20, 45.33]	0.608
Tidal volume, ml [IQR]	362 [280, 499]	395 [275, 510]	0.332
Septic shock, *n* (%)	188 (62.7)	89 (63.1)	1.000
Lactate, mg/dL [IQR]	18.00 [11.00, 47.75]	21.00 [11.00, 48.00]	0.542
RRT, *n* (%)	196 (65.3)	95 (67.4)	0.747
Inhaled nitric oxide, *n* (%)	218 (72.7)	110 (78.0)	0.244
Prone positioning, *n* (%)	222 (74.0)	108 (76.6)	0.638
Mean ECMO sweep gas flow, L/min [IQR]	3.86 [2.60, 5.14]	6.04 [4.43, 7.66]	<0.001
Mean ECMO Q̇_EC_, L/min [IQR]	3.16 [2.72, 3.56]	4.59 [4.20, 4.96]	<0.001
ECMO duration (days)	12.00 [7.00, 26.00]	17.00 [7.00, 28.00]	0.371
PRBC units transfused, number [IQR]	17.00 [8.00, 32.25]	25.00 [13.00, 40.00]	0.001
Mean Hp	0.62 [0.33, 1.20]	0.39 [0.21, 0.86]	<0.001
Mean CFH	8.12 [5.44, 13.75]	10.64 [6.42, 19.25]	0.001
ICU mortality	118 (39.3)	101 (71.6)	<0.001

Definition of abbreviations: Q̇_EC_ = blood flow, IQR = interquartile range, SOFA = Sequential Organ Failure Assessment, APACHE = Acute Physiology And Chronic Health Evaluation, SAPS = Simplified Acute Physiology Score, RASS = Richmond Agitation–Sedation Scale, ARDS= Acute Respiratory Distress Syndrome, PIP = peak inspiratory pressure, Pplat = plateau pressure, PEEP = positive end-expiratory pressure, RRT = renal replacement therapy, ECMO = extracorporeal membrane oxygenation, PRBC = packed red blood cells, CFH = cell-free hemoglobin, Hp = Haptoglobin, ICU = intensive care unit. Data are expressed as mean (SD), median (25%, 75% quartiles) or frequencies (%), as appropriate.

**Table 2:** Multivariate regression model for ICU mortality.

Variable	OR [95%CI]	*p* Value
ECMO Q̇_EC_ (L/min)	1.43 [1.09–1.88]	0.010
Mean CFH (mg/dL)	1.06 [1.03–1.08]	<0.001
Mean Hp (g/L)	0.50 [0.34–0.70]	<0.001
Age (year)	1.04 [1.02–1.06]	<0.001
Male sex	0.57 [0.34–0.94]	0.028
PRBCs	1.00 [0.99–1.01]	0.73
ECMO sweep gas flow (L/min)	1.43 [1.25–1.65]	<0.001

Definition of Abbreviations: OR—odds ratio, CI—confidence interval, Q̇_EC_—blood flow, CFH—cell-free hemoglobin, Hp—Haptoglobin, PRBC—packed red blood cells, ECMO = extracorporeal membrane oxygenation.

## Data Availability

Data are available from the corresponding author on reasonable request.
